# Cytochrome P450 *CYP1B1* Interacts with 8-*Methoxypsoralen* (8-MOP) and Influences Psoralen-Ultraviolet A (PUVA) Sensitivity

**DOI:** 10.1371/journal.pone.0075494

**Published:** 2013-09-23

**Authors:** Yusuf Y. Deeni, Sally H. Ibbotson, Julie A. Woods, C. Roland Wolf, Gillian Smith

**Affiliations:** 1 Division of Cancer Research, Medical Research Institute, University of Dundee, Dundee, United Kingdom; 2 Photobiology Unit, University of Dundee, Dundee, United Kingdom; 3 Cancer Research UK Molecular Pharmacology Unit, Ninewells Hospital and Medical School, Dundee, United Kingdom; 4 School of Contemporary Science, University of Abertay Dundee, Dundee, United Kingdom; Tor Vergata University of Rome, Italy

## Abstract

**Background:**

There are unpredictable inter-individual differences in sensitivity to psoralen-UVA (PUVA) photochemotherapy, used to treat skin diseases including psoriasis. Psoralens are metabolised by cytochrome P450 enzymes (P450), and we hypothesised that variability in cutaneous P450 expression may influence PUVA sensitivity. We previously showed that P450 *CYP1B1* was abundantly expressed in human skin and regulated by PUVA, and described marked inter-individual differences in cutaneous *CYP1B1* expression.

**Objectives:**

We investigated whether *CYP1B1* made a significant contribution to 8-methoxypsoralen (8-MOP) metabolism, and whether individuality in *CYP1B1* activity influenced PUVA sensitivity.

**Methods:**

We used *E. coli* membranes co-expressing various P450s and cytochrome P450 reductase (*CPR*) to study 8-MOP metabolism and cytotoxicity assays in *CYP1B1*-expressing mammalian cells to assess PUVA sensitivity.

**Results:**

We showed that P450s *CYP1A1, CYP1A2, CYP1B1, CYP2A6* and *CYP2E1* influence 8-MOP metabolism. As *CYP1B1* is the most abundant P450 in human skin, we further demonstrated that: (i) *CYP1B1* interacts with 8-MOP (ii) metabolism of the *CYP1B1* substrates 7-ethoxyresorufin and 17-β-estradiol showed concentration-dependent inhibition by 8-MOP and (iii) inhibition of 7-ethoxyresorufin metabolism by 8-MOP was influenced by *CYP1B1* genotype. The influence of *CYP1B1* on PUVA cytotoxicity was further investigated in a Chinese hamster ovary cell line, stably expressing *CYP1B1* and *CPR*, which was more sensitive to PUVA than control cells, suggesting that *CYP1B1* metabolises 8-MOP to a more phototoxic metabolite(s).

**Conclusion:**

Our data therefore suggest that *CYP1B1* significantly contributes to cutaneous 8-MOP metabolism, and that individuality in *CYP1B1* expression may influence PUVA sensitivity.

## Introduction

Psoralen-UVA (PUVA) photochemotherapy is widely used to treat psoriasis and other common skin diseases [1], [2] . Importantly, PUVA is of superior efficacy to some of the highly costly biologic drugs, with unknown long-term safety, that are increasingly being used to treat psoriasis [3]. There is considerable individuality in PUVA sensitivity, and high cumulative PUVA exposure significantly increases risk of skin cancer [4]. To date, no reliable predictors of PUVA sensitivity have been identified, although there is increasing interest in genetic response predictors [5]. Knowledge of genetic or phenotypic determinants of PUVA sensitivity would facilitate the identification of individuals at increased risk of acute or chronic adverse effects of PUVA treatment.

The most commonly clinically used psoralen is the furanocoumarin, 8-methoxypsoralen (8-MOP), used in systemic and topical PUVA therapy. Previous studies have shown correlations between serum 8-MOP concentrations and PUVA sensitivity, assessed by minimal phototoxic dose (MPD), although there is considerable inter-individual variability in 8-MOP serum levels following oral administration [6]–[9]. This individuality may reflect inter-individual differences in 8-MOP metabolism, but this also suggests that variation in hepatic P450 gene expression may not be the only contributor and that cutaneous drug metabolising enzymes (DME) may influence PUVA sensitivity. There is considerable evidence that variation in DME, in particular cytochrome P450, activity can be a major determinant of individuality in drug sensitivity [10], [11]. In support of this hypothesis, we have shown that PUVA induces the cutaneous expression of several P450s, and reported marked inter-individual variability in constitutive and inducible P450 expression [12], [13].

P450s are a multigene family of inducible Phase I DMEs which, together with their redox partner NADPH cytochrome P450 reductase (*CPR*), catalyse the oxidative metabolism of many endogenous compounds, drugs and xenobiotics [14]–[16]. In general, compounds which are P450 inducers are also P450 substrates or inhibitors [17]. P450s have been implicated in psoralen metabolism in insects [18], [19] and vertebrates [20], where different furanocoumarin derivatives induce or inhibit multiple P450s. In contrast, human P450s involved in 8-MOP metabolism have not been fully characterised, although furanocoumarins have been shown to inhibit human *CYP1A2* [21] and *CYP1B1* [22], polymorphisms in *CYP2A13* shown to influence the metabolism of 5-methoxypsoralen [23] and the furanocoumarin chalepsin identified as both a substrate and inhibitor of coumarin hydroxylase (*CYP2A6*) [24].

We previously showed that the predominantly extra-hepatic *CYP1B1* was the most abundantly expressed P450 in human skin, with marked inter-individual variability in both constitutive expression and in regulation by UVR and PUVA [12]. Pharmacogenetic polymorphisms result in a population distribution of alleles of many P450 including *CYP1B1* (CYP1B1*1 to CYP1B1*26, http://www.cypalleles.ki.se/cyp1b1.htm *)* resulting in altered gene expression and catalytic activity. We therefore investigated whether *CYP1B1* metabolises 8-MOP, and whether individuality in *CYP1B1* expression influences PUVA sensitivity. Our data suggest that *CYP1B1* significantly contributes to cutaneous 8-MOP metabolism, and that individuality in *CYP1B1* expression may influence PUVA sensitivity.

## Materials and Methods

### Ethical Approval

The use of human skin samples was approved by the Tayside Committee on Medical Research Ethics (091/99). All patients were given a patient information sheet detailing the study and given at least 24 h to digest this before providing written informed consent on a standard consent form approved by the Tayside Committee on Medical Research Ethics.

### Co-expression of Human *CYP1B1* and *CPR* in *E. coli*

Construction of *CYP1B1* and *CPR* expression plasmids has been described previously [25]. The *CYP1B1* alleles CYP1B1*1, CYP1B1*3 and CYP1B1*4 were co-expressed with *CPR* in *E. coli JM109* cells and membranes prepared as described previously [26]. P450 content was determined spectrophotometrically [27], and *CPR* activity estimated by cytochrome *c* reduction [26]. 8-MOP metabolism was assessed by HPLC analysis, as previously described [28]. Western blot analysis was performed after cells were pre-incubated with 0.05% PBS–EDTA, harvested by trypsinisation (0.25% trypsin-EDTA) and protein content assessed by Bradford assay [29]. Solubilised protein lysates or human liver microsomes were separated by SDS-PAGE, transferred onto Hybond-ECL nitrocellulose membranes (Amersham, UK) and probed with *CYP1B1* (BD Gentest, Woburn, MA) and *CPR* antibodies [30], as previously described [25].

### Interaction of 8-MOP with Recombinant P450s

Furanocoumarin metabolism was assessed using a substrate depletion assay, as previously described [31], [32]. Assays were performed with 5 µM 8-MOP in 160 µl reaction mixtures containing 50 pmol recombinant P450 membranes or 250 µg human liver microsomes (Tayside Tissue Bank, positive controls) in 50 mM phosphate buffer (PB) pH 7.4. After 3 min 37°C pre-incubation, reactions were initiated by 40 µl 5 mM NADPH containing 30 mM glutathione (GSH) in 50 mM PB (pH 7.4). Reactions were incubated for 10 min in a shaking water bath at 37°C, terminated by the addition of 100 µl ice-cold methanol containing 3% perchloric acid, and placed on ice for 10 min. Zero-time controls were terminated before the addition of P450 membranes; no-NADPH control reactions were initiated with 20 µl 50 mM PB (pH 7.4) containing 30 mM GSH. Samples were vortexed for 1 min, centrifuged at 12 000 rpm for 5 min, supernatants collected and analysed by HPLC analysis [28].

### The Influence of 8-MOP on Recombinant Human *CYP1B1* Activity

The role of *CYP1B1* in 8-MOP metabolism was confirmed using 17-β-estradiol 2-hyroxylase, 4-hydroxylase and 7-ethoxyresorufin O-deethylase (EROD) assays. Estradiol hydroxylase assays were performed as previously described [25] using 20 µM 17-β-estradiol in the absence (methanol solvent control) or the presence of 8-MOP (1, 10, or 100 µM). EROD activity was determined as previously described [33], but modified in 96-well plate format with quadruplicate replicates, using a Fluoroskan fluorescence reader (Labsystems, Cambridge, UK). Each 200 µl incubation mixture contained PBS, 4 µM 7-ethoxyresorufin, 250 µM NADPH, 5 pmol recombinant *CYP1B1*, and 8-MOP (0–180 µM, serially diluted). Assays were performed at 37°C, fluorescence readings recorded every 20 s for 3 min at λ_ex_ = 530 nm, λ_em_ = 584 nm and fluorescence production (arbitrary fluorescence units) against time relative to control incubations used to assess 8-MOP inhibition. Calibration curves were constructed with authentic resorufin standards and linear regression analysis used to calculate the amount of resorufin formed in each test sample. Enzyme kinetics for *CYP1B1*-catalysed EROD activity were determined at substrate concentrations from 0.002 to 2 µM. Apparent K_m_ and V_max_ values were estimated by non-linear regression analysis (GraFit software program, UK) of the substrate concentration [S]-enzyme activity [V], fitting data to a Michaelis-Menten model. Similarly, the concentration of 8-MOP required to inhibit 50% (IC_50_) of *CYP1B1*-catalysed EROD at K_m_ concentration was calculated. Experiments were performed using six 8-MOP concentrations 8-MOP (0, 1.17, 2.34, 4.69, 9.38 and 37.50 µM) and four 7-ethoxyresorufin concentrations equivalent to 1/2 K_m_, K_m_, 2 K_m_ and 5 K_m_ - mode of inhibition was determined by Lineweaver-Burk plots.

### Stable Expression of Human *CYP1B1* and *CPR* in CHO Cells

CHO cell lines stably over-expressing *CYP1B1* and *CPR* were generated as previously described [34]. CHO cells were maintained at 37°C/5% CO_2_ in Dulbecco’s Modified Eagle’s medium (DMEM) supplemented with 10% fetal bovine serum (FBS), 100 U/ml penicillin, 100 µg/ml streptomycin, 1 U/ml each of hypoxanthine and thymine (Invitrogen, Paisley-UK). Similar culture conditions, with the addition of G-418 (400 µg/ml), were used for CHO cells stably expressing *CPR* (CHO-*CPR*). CHO cells stably co-expressing *CYP1B1* and *CPR* (CHO-*1B1/CPR*) were maintained at 37°C/5% CO2, but in hypoxanthine and thymine-free DMEM supplemented with 10% dialysed FBS and the appropriate selection (0.3 µM MTX and 400 µg/ml G-418). HaCaT keratinocytes were grown to 80% confluence in DMEM supplemented with 10% fetal bovine serum (FBS), 100 U/ml penicillin, 100 µg/ml streptomycin and 2 mM glutamine. Cells were harvested and microsomes prepared as described previously [26]. HaCaT cell microsomes and microsomes from human skin biopsies obtained from peri-lesional skin from psoriasis patients [12], [13] were used to investigate functional CYP1B1 expression using EROD assays.

### UVA and PUVA Treatment

To investigate CHO cell sensitivity to UVA and PUVA, actively dividing cells (10000 cells/well) were seeded in 96-well plates (Iwaki, Japan) in quadruplicates in 200 µl culture medium and allowed to grow for 18–20 h. Cells were irradiated at room temperature with a UVA source (2×6 ft Cosmolux 15500/100 W, 1.7 mW/cm^2^ (320–400 nm; λmax = 365 nm), Germany) through a 4 mm thick window glass filter at multiple UVA doses (0, 1.5, 4.0 and 7.5 J/cm^2^), monitoring irradiance with a Waldmann UV meter equipped with UVA detector. Control samples were kept in the dark under the same conditions. To model PUVA treatment, cells were pre-incubated with varying concentrations of 8-MOP (0–5 µM) in PBS at 37°C for 40 min and cells irradiated as described above. Control samples were pre-incubated with solvent and kept in the dark under the same conditions. After each treatment, fresh culture medium was added and cells returned to an incubator at 37°C/5%CO_2_ for 24 h. Cell viability was assessed using neutral red uptake assays [35]; quadruplicate replicates of each treatment were performed, and each experiment repeated twice. PUVA cytotoxicity data was assessed by non-linear regression analysis for dose-response curves (IC_50_) fitting (GraFit software program, UK) and unpaired Student’s *t*-test (Statview 4.1 software).

### Tissue Immunohistochemistry

To confirm *CYP1B1* and *CPR* protein expression in human skin, frozen non-lesional skin biopsies from patients with psoriasis, and from whom informed consent was obtained and approved by Tayside Committee on Medical Research Ethics, were analysed by immunohistochemistry using polyclonal antibodies against human *CYP1B1* (BD Gentest, Woburn, MA) and human *CPR* [25], [30]. 4 µm frozen sections were cut from 4 mm full-thickness punch biopsies and processed in a standard immunohistochemical procedure with microwave antigen retrieval and horseradish-peroxidase-labelled streptavidin detection system and counterstained with haematoxylin-eosin.

## Results

### 8-MOP Metabolism by Recombinant P450s

Human liver microsomes from three different individuals expressing multiple human P450s (positive controls) and *E. coli* membranes co-expressing individual P450s present in human skin (*CYP1A1, CYP1A2, CYP1B1, CYP2A6, CYP2E1*) and *CPR* were used to identify P450s involved in 8-MOP metabolism. 8-MOP was metabolized by each of three human liver microsomes, with specific activities of 1.31, 3.53 and 9.35 pmol/min/mg protein, respectively (mean ± SD; 4.73±4.15, Table 1). Each recombinant P450 metabolised 8-MOP in the presence of *CPR* (Table 1), but not in reactions containing *CPR* alone or in control experiments omitting *CPR* or the essential cofactor NADPH (data not shown). 8-MOP metabolism in human liver microsomes and by recombinant *CYP1A1, CYP1A2* and *CYP1B1* was decreased in the presence of the potent, selective CYP1 P450 family inhibitor α-naphthoflavone (5 µM), where microsomal, *CYP1A1-, CYP1A2-* and *CYP1B1*-dependent 8-MOP metabolism was reduced to 59, 53, 34 and 62% of control levels, respectively. Of the catalytically active P450s tested, *CYP1B1* is most abundantly expressed in human skin [12], and was therefore selected as the focus of additional studies.

**Table 1:** Metabolism of 8-MOP by human recombinant P450s and human liver microsomes^a^.

P450	Turnover (min^−1^)
*CYP1A1*	0.29±0.07
*CYP1A2*	0.63±0.08
*CYP1B1*	0.24±0.05
*CYP2A6*	0.48±0.04
*CYP2E1*	0.08±0.03
*CPR*	ND^c^
Microsomes^b^	4.73±4.15

a Assays were performed with 5 µM 8-MOP as described in Materials and Methods. Each value represents mean ± SD of three determinations.

b Mean ± SD of specific activity (n = 3) of microsomes from 3 human livers as pmol/min/mg protein.

c ND; Not detected.

### 8-MOP Inhibits *CYP1B1* Activity

**Figure 1:**
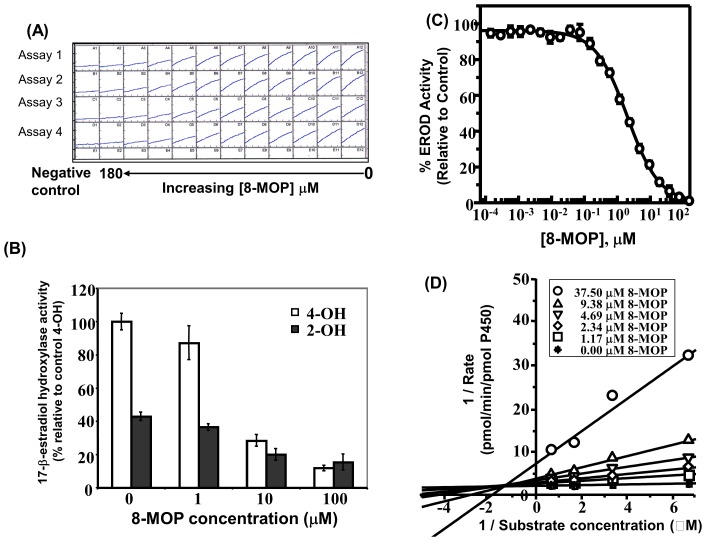
8-MOP influences CYP1B1 catalytic activity. The influence of 8-MOP on: (A) EROD activity and (B) 17-β-estradiol hydroxylase activity in *E. coli* membranes co-expressing *CYP1B1* and CPR. 17-β-estradiol 4-hydroxylase and 17-β-estradiol 2-hydroxylase activities were determined as described in Materials and Methods and are presented as mean ± SD of % control 17-β-estradiol 4-hydroxylation. (C) Dose-response curve for 8-MOP dependent *CYP1B1* inhibition. EROD activity was assessed as described in Materials and Methods and data presented as mean ± SD percentage of control activity of three independent determinations. (D) Lineweaver-Burk plots for inhibition of *CYP1B1* activity by 8-MOP. *CYP1B1* catalysed EROD activity was determined at varying concentrations of 8-MOP and 7-ethoxyresorufin, as described in Materials and Methods. Data represent linear regression analysis of the mean of transformed data for three independent determinations.

Using *E. coli* membranes co-expressing recombinant *CYP1B1* and *CPR*, we showed that 8-MOP inhibited *CYP1B1*-dependent EROD and 17-β-estradiol hydroxylase activities in a concentration dependent manner (Figures 1A & 1B). The apparent K_m_ for *CYP1B1*-dependent EROD activity was 0.33±0.03 µM (n = 3), consistent with previous literature [25]. The addition of 8-MOP decreased *CYP1B1*-dependent EROD activity in a concentration-dependent manner with an estimated IC_50_ of 2.0±0.1 µM (Figure 1C) and Ki of 3.58±1.00 µM (n = 3). Further, the IC_50_ for 8-MOP metabolism by the low activity CYP1B1*4 allele was significantly reduced relative to the “wild-type” CYP1B1*1, and CYP1B1*3 alleles (Table 2).

**Table 2:** CYP1B1*4 influences 8-MOP metabolism.

*CYP1B1* allelic variant	8-MOP Turnover	IC_50_ a	
	(min^−1^)	(ng/ml)	( µM)
CYP1B1*1	0.27±0.05	369±43	1.7±0.20
CYP1B1*3	0.25±0.03	326±15	1.5±0.07
CYP1B1*4	0.12±0.02	130±22	0.6±0.10

a IC_50_ for 8-MOP against *CYP1B1-*dependent EROD activity determined using *E. coli* membranes co-expressing human individual *CYP1B1* alleles and *CPR* as described in Materials and Methods. Data represents the mean ± SD of triplicate determinations.

To determine the potency and mode of 8-MOP inhibition of *CYP1B1*, further experiments were performed using six 8-MOP concentrations and four 7-ethoxyresorufin concentrations as described in Materials and Methods. Analysis of enzyme kinetic data by Lineweaver-Burk plots suggested that 8-MOP inhibited *CYP1B1* by mixed inhibition (Figure 1D).

### Evidence for the Presence of Catalytically Active *CYP1B1* in Human Skin

**Figure 2:**
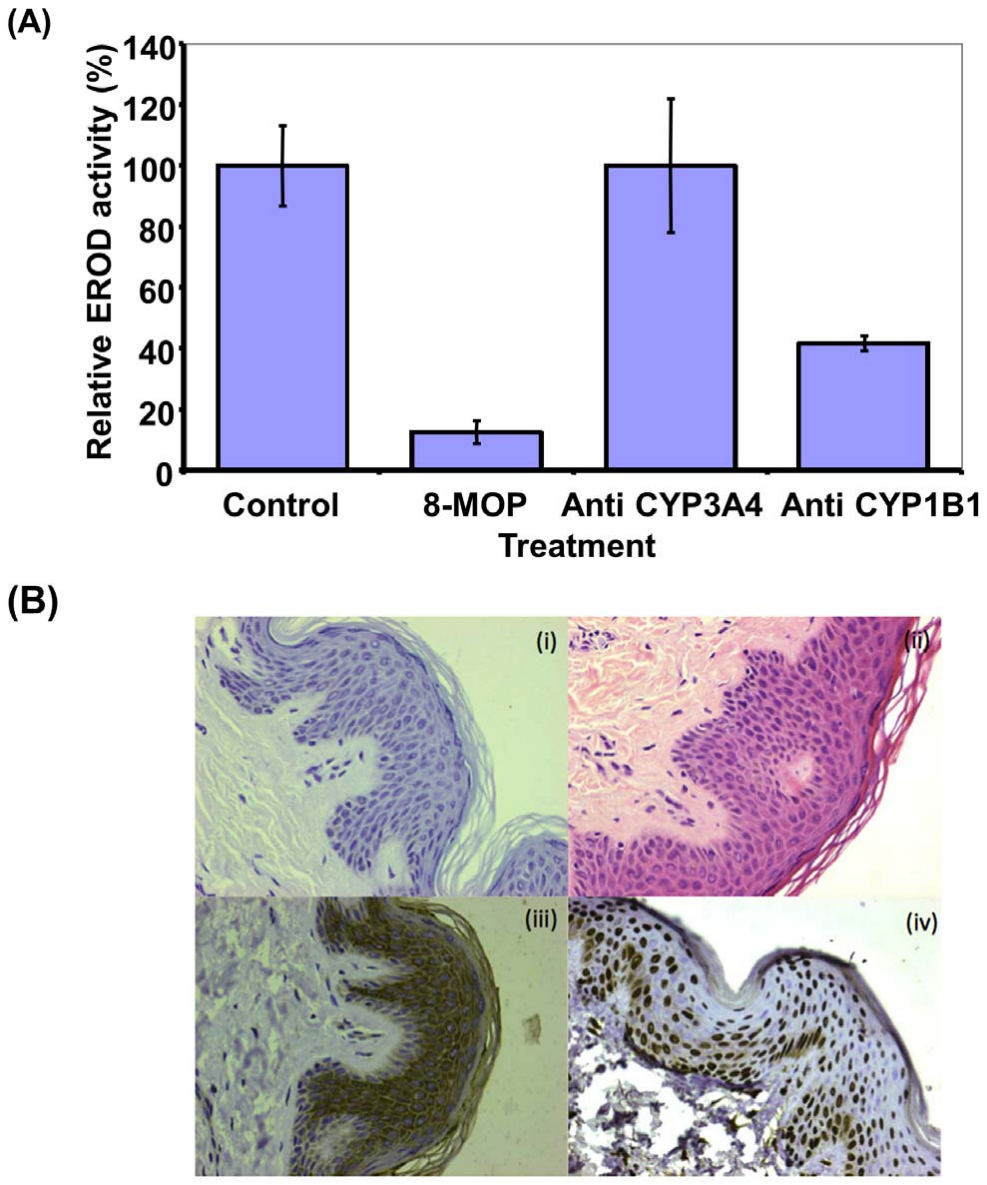
The presence of catalytically active *CYP1B1 in human skin*. (**A**) Inhibition of EROD activity by 8-MOP and *CYP1B1* polyclonal antibodies in HaCaT keratinocyte microsomes. EROD activity was determined as described in Materials and Methods. Pre-incubation was performed with 8-MOP (5 µM), and anti-*CYP1B1* and anti-*CYP3A4* polyclonal antibodies as described in Materials and Methods. (**B**) Cytochrome P450 *CYP1B1* and *CPR* expression in non-lesional skin of patients with psoriasis was assessed as described in Materials and Methods. (**i**) Negative (no antibody) control (**ii**) Hematoxylin-Eosin stain (**iii**) *CYP1B1* antibody (1∶1000) stain and (**iv**) *CPR* antibody (1∶2000) stain.

EROD activity was determined in microsomes isolated from human skin and HaCaT keratinocytes, with turnover rates of 0.10±0.05 and 0.30±0.03 pmol/min/mg protein, respectively. EROD metabolism was significantly reduced in the presence of 5 µM 8-MOP (90% inhibition), and following pre-incubation of HaCaT microsomes with a *CYP1B1*-specific antibody (60% inhibition) (Figure 2A). In contrast, incubation of microsomes with control *CYP3A4* antibodies had no effect on EROD activity. Consistent with our previous data, cutaneous *CYP1B1* and *CPR* expression was confirmed by immunohistochemistry (Figure 2B). Nuclear CPR localization in human skin was unexpected and may reflect a tissue fixation or processing artefact.

### *CYP1B1* and *CPR* Over-expression Influences PUVA Sensitivity in CHO Cells

**Figure 3:**
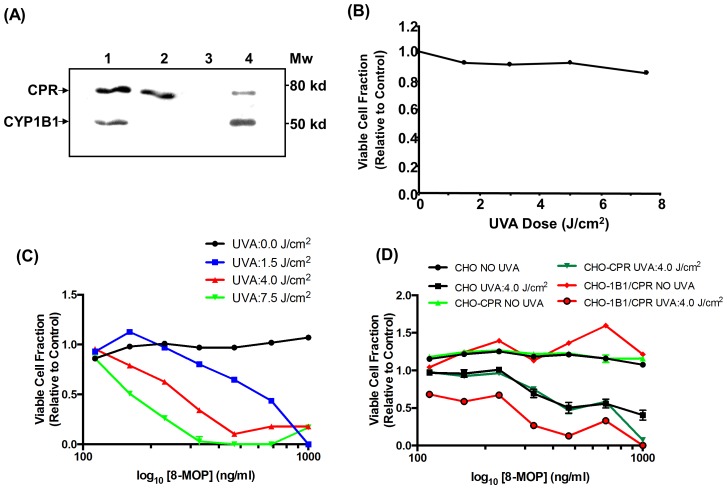
CYP1B1 expression in CHO cells influences psoralen-UVA sensitivity. (**A**) Western blot analysis of *CYP1B1* and *CPR* expression in modified CHO cell lines was performed as described in Materials and Methods (lane 1, CHO-1B1/CPR cells; lane 2, CHO-*CPR* cells; lane 3, CHO cells; lane 4, control *E*. *coli* membranes). The influence of (**B**) UVA and (**C**) PUVA treatment on CHO cell viability and (**D**) PUVA treatment on CHO-*1B1*/*CPR* and CHO-*CPR* cells was assessed by NRUA [35]. Data are presented from representative experiments and expressed as mean ± SD of duplicate determinations compared to dark control (for the majority of cell lines, experimental errors were very small and SD values are therefore too small to be visible).

We have previously reported that CHO cells have no constitutive P450 and limited *CPR* expression, but have been engineered to stably over-express *CYP1B1* and *CPR*, individually and in combination [34] (Figure 3A). As described in Materials and Methods, the presence of catalytically active *CYP1B1* was confirmed using EROD assays in total protein cell lysates from each cell line. While there was no EROD activity in control CHO or CHO-*CPR* expressing cells, CHO-*1B1/CPR* expressing cells metabolised ethoxy resorufin (EROD activity 1.85±0.87 pmol/min/mg protein; n = 5).

UVA alone at doses up to 7.5 J/cm^2^ and 8-MOP (0–5 µM) alone showed no significant cytotoxicity to CHO cells (Figure 3B & 3C). However, significant cytotoxicity was seen when CHO cells were exposed to 8-MOP and then irradiated with UVA, with dose-dependent effects of both 8-MOP concentration and UVA dose (Figure 3C). Optimal dose-dependency cytotoxicity was seen at a combined UVA dose of 4 J/cm^2^ and an 8-MOP concentration range of 0–5 µM (Figure 3C).

8-MOP alone was not cytotoxic and appeared to promote cell proliferation in each cell line tested, although this was most evident for the catalytically-active CHO-*1B1/CPR* cell line (Figure 3D). In contrast, PUVA was cytotoxic to all the cell lines tested (Figure 3D). While the cytotoxic effect of PUVA on the CHO and the CHO-*CPR* cell lines were similar (p = 0.268, Table 3), the CHO-*1B1/CPR* cell line was significantly more sensitive to PUVA cytotoxicity than the parental CHO cell line (p = 0.002) or the CHO-*1B1/CPR* cell line (p<0.001). The addition of catalytically active *CYP1B1* to CHO cells resulted in an approximately 2.6-fold increase in PUVA phototoxicity.

**Table 3:** Modulation of PUVA cytotoxicity in CHO cells by *CYP1B1* expression.

Cell line	IC_50_ a		
	(ng/ml)	( µM)	p-Value
CHO	461±47	2.13±0.22	
CHO-*CPR*	540±48	2.50±0.22	0.268^b^
CHO-*1B1/CPR*	241±23	1.11±0.11	0.002^b^<0.001^c^

a IC_50_ for 8-MOP in the presence of UVA (4 J/cm^2^) determined as described in Materials and Methods. Data represents the mean ± SE of 5 experiments.

b p-value as compared with control CHO cell line.

c p-value as compared with CHO-*CPR* cell line.

## Discussion

Psoralens, particularly systemic and topical 8-MOP, are widely used in dermatology in PUVA photochemotherapy. However, the enzymes involved in 8-MOP metabolism have not been fully characterised, and it is not known whether individuality in 8-MOP metabolism may influence PUVA sensitivity. Previous studies have described O-demethylation, hydroxylation and epoxidation as important mechanisms of 8-MOP metabolism [6], [36]. This, together with the observation that phenobarbital and α-naphthoflavone induce 8-MOP metabolism *in vivo* and *in vitro* [36], suggest a role for P450s in 8-MOP metabolism, and an inhibitory effect of furanocoumarins on human *CYP1A2* has recently been confirmed [21]. However, with the exception of the predominantly hepatic CYP2A6 [37], [38] and respiratory tract-specific *CYP2A13* [23], [39], cutaneous P450s active in 8-MOP metabolism have not been characterised.

We have previously shown that the cutaneous expression of several P450s, including *CYP1B1, CYP2S1* and *CYP2E1,* is regulated by PUVA *in vivo,* and have shown marked individuality in gene expression [12]. *CYP1B1* is the most abundantly expressed P450 in human skin, and furanocoumarins have been shown to inhibit *CYP1B1* activity [22] . 8-MOP is highly structurally similar to the *CYP2A6* substrate coumarin, another competitive *CYP1B1* inhibitor [40], suggesting that cutaneous *CYP1B1* expression may influence PUVA sensitivity. We found that 8-MOP inhibits *CYP1B1*-dependent EROD and 17-β-estradiol hydroxylase activities in a concentration-dependent manner, with estimated IC_50_ values for EROD similar to those reported for another furanocoumarin, bergamottin [41]. Moreover, the inhibition of *CYP1B1*-dependent EROD metabolism by 8-MOP was demonstrated in both human skin microsomes and in a human keratinocyte cell line. These data suggest that 8-MOP may be a *CYP1B1* substrate and demonstrate that 8-MOP and/or its metabolite(s) are potent *CYP1B1* inhibitors.

The inhibition of *CYP1B1* by 8-MOP and/or metabolites could occur by direct selective inhibition and/or mechanism-based inactivation as reported with other P450s [21]. Previously, 8-MOP and other furanocoumarin derivatives have been shown to be potent mechanism-based P450 inhibitors [38], [42], with the generation of a reactive intermediate by initial oxidation of the furan ring to form furanoepoxide intermediates that readily react with target residue(s) in the active site of the P450 [24]. Currently, it is not known whether 8-MOP inhibits the catalytic activity of *CYP1B1 in vivo*, however clinical data suggest that this is likely [37], [43].

We were therefore interested to find that, using a modified CHO cell line stably over-expressing recombinant human *CYP1B1* together with its redox partner *CPR*, CHO-1B1/*CPR* cells were significantly more susceptible to PUVA cytotoxicity than control cell lines. These data strongly suggest that *CYP1B1* may metabolise 8-MOP to product(s) which are more phototoxic than the parent compound. In support of this hypothesis, we have recently shown that CYP1B1 is induced by UVB, but is not significantly induced by UVA in HaCaT keratinocytes [29], consistent with increased PUVA sensitisation arising from altered 8-MOP metabolism. Evidence from several cell line models additionally suggests that PUVA is a source of reactive oxygen species leading to oxidative stress [44], genotoxic effects and the formation of DNA photoadducts [45]. It is therefore conceivable that the increased PUVA cytotoxicity in the CHO*-1B1/CPR* cell line may reflect increased genotoxicity and DNA photoadduct formation and this requires further study.

*CYP1B1* is polymorphic, with a population distribution of alleles with altered catalytic activity [46]. In particular, the CYP1B1*3 allele (Val_432_Leu) allele has been shown to significantly influence the conversion of estradiol to 4-hydroxyestradiol [25] and the CYP1B1*4 allele (Asn_453_Ser) to have reduced protein expression and catalytic activity, as a consequence of enhanced polyubiquitin-mediated degradation [47]. Our finding that 8-MOP metabolism is also impaired in cells expressing the CYP1B1*4 allele is therefore intriguing, and suggests that both genetically determined and transcriptionally regulated inter-individual differences in *CYP1B1* activity may influence PUVA sensitivity. As PUVA is photocarcinogenic, with high cumulative PUVA exposure incurring a significantly increased risk of skin cancer [4], it will be of particular interest in future clinical studies to investigate whether CYP1B1 genotype influences PUVA-associated skin cancer risk, and whether *CYP1B1* genotype or phenotype influences PUVA sensitivity.
